# Bryophyte-Feeders in a Basal Brachyceran Lineage (Diptera: Rhagionidae: Spaniinae): Adult Oviposition Behavior and Changes in the Larval Mouthpart Morphology Accompanied with the Diet Shifts

**DOI:** 10.1371/journal.pone.0165808

**Published:** 2016-11-03

**Authors:** Yume Imada, Makoto Kato

**Affiliations:** Graduate School of Human and Environmental Studies, Kyoto University, Yoshidanihonmatsu-cho, Sakyo-ku, Kyoto, 6068501, Japan; Smithsonian National Museum of Natural History, UNITED STATES

## Abstract

Dipteran larval morphology exhibits overwhelming variety, affected by their diverse feeding habits and habitat use. In particular, larval mouthpart morphology is associated with feeding behavior, providing key taxonomic traits. Despite most larval Brachycera being carnivorous, a basal brachyceran family, Rhagionidae, contains bryophyte-feeding taxa with multiple feeding habits. To elucidate the life history, biology, and morphological evolution of the bryophyte-feeding rhagionids, the larval feeding behavior and morphology, and the adult oviposition behavior of four species belonging to three genera of Spaniinae (*Spania* Meigen, *Litoleptis* Chillcott and *Ptiolina* Zetterstedt) are described. Moreover, changes of the larval morphology associated with the evolution of bryophyte-feeding are traced by molecular phylogenetic analyses. *Spania* and *Litoleptis* (thallus-miners of thallose liverworts) share a toothed form of apical mandibular sclerite with an orifice on its dorsal surface, which contrasts to those of the other members of Rhagionidae possessing a blade-like mandibular hook with an adoral groove; whereas, *Ptiolina* (stem borer of mosses) exhibits a weak groove on the adoral surface of mandible and highly sclerotized maxilla with toothed projections. Based on the larval feeding behavior of the thallus-miners, it is inferred that the toothed mandibles with the dorsal orifice facilitate scraping plant tissue and then imbibing it with a great deal of the sap. A phylogeny indicated that the bryophyte-feeding genera formed a clade with *Spaniopsis* and was sister to *Symphoromyia*, which presumably are detritivores. This study indicates that the loss or reduction of adoral mandibular groove and mandibular brush is coincident with the evolution of bryophyte-feeding, and it is subsequently followed by the occurrence of dorsal mandibular orifice and the loss of creeping welts accompanying the evolution of thallus-mining.

## Introduction

Diptera is one of the four megadiverse insect orders, with the larvae of this order inhabiting a diverse range of environments. Flies have experienced dynamic and recurrent shifts in habitat type and food availability [1], associated with major changes in larval morphology [2]. Brachycera originated and radiated during the Late Triassic [3]. During this time, the mandibles of the larvae noticeably changed from horizontal to vertical mobility [3–5], correlated with an evolutionary trend of feeding habit from saprophagy to predation [3]. As such the larval mouthpart morphology reflects the evolution of diet, and has been effectively employed as key characters in tracing the intricate evolutionary history of Diptera [5–9].

Carnivory is the primary, and presumably plesiomorphic, larval feeding habit of Brachycera [3]. Phytophagous feeding habits are known in at least 34 families of Diptera [10]. In early lineages of Brachycera (i.e. Orthorrhapha), herbivory occurs in just two families, Dolichopodidae [11] and Rhagionidae [12–15]. Herbivorous species of Rhagionidae feed on bryophytes [12–15]; however, knowledge of larval feeding habits remains limited.

Rhagionidae is one of the most plesiomorphic brachyceran lineages [1, 9, 16, 17], containing about 700 extant species [18] and more than 80 fossil species [19, 20]. Although the biology of rhagionid larvae is poorly known, multiple feeding habits have been documented. The biology of six out of the 16 genera has been documented to date [18]. The larvae of *Rhagio* Fabricius are found in damp soil under shallow leaf carpets and are predatory, feeding on earthworms and soft-bodied insect larvae [4, 21]. *Chrysopilus* Macquart contains predators [21] and rotten wood feeders [22, 23], which occur in a broad range of habitats in terrestrial and aquatic environments [23–25]. *Symphoromyia* Frauenfeld tend to be detritivores or to feed on decayed plant materials in damp soil [26]. The phytophagous feeding habit has been detected in three rhagionid genera. Specifically, *Spania* Meigen and *Litoleptis* Chillcott are thallus-miners of liverworts [12, 13, 15], while *Ptiolina* Zetterstedt is a grazer of mosses [14, 27, 28] and liverworts [13].

The bryophyte-feeding genera have been assigned to the subfamily Spaniinae, together with *Omphalophora* Becker, *Spaniopsis* White, and *Symphoromyia* [15, 18]. Spaniinae is one of three subfamilies of Rhagionidae defined on the basis of female genital morphology [15, 18, 29, 30], and its monophyly is well-supported in a phylogenetic analysis based on morphological and molecular data [18]. Therefore, it is likely that the bryophyte-feeding had evolved within Spaniinae, although the phylogenetic relationships among spaniine genera are still unclear.

The larval morphology of Rhagionidae is often hypothesized to represent the ground plan of Brachycera [4, 8]. Four out of 16 genera have been described to date; namely, *Chrysopilus* [22, 23], *Rhagio* [21], *Symphoromyia* [26], and *Ptiolina* [18]. The larval morphology of Rhagionidae is reviewed in [17], with close examinations of 14 morphological characters for 20 genera of Tabanomorpha. The known rhagionid larvae have suctorial mouthparts [2, 4, 25], with a blade-like mandibular hook that is suited for the predatory feeding habit [4, 27]. Nevertheless, knowledge on the bryophyte-feeding rhagionids, particularly on the thallus-mining genera, remains largely unknown. Thus, some of the morphological characteristics that have led to herbivory in Rhagionidae will be evident in the larval mouthparts morphology by identifying and comparing those of herbivorous taxa with non-herbivorous taxa.

In this study, the biology, larval morphology and adult oviposition behavior of the three bryophyte-feeding genera are described, based on detailed field observation and morphological examination. Specifically, the larval mouthpart structures of the bryophyte-feeders are emphasized, which are strikingly different from those of all other Tabanomorpha. The functional morphology of the mouthparts is inferred, which is further validated from direct observations of feeding behavior. These data on the mouthpart morphology are combined with the molecular phylogeny of Spaniinae, thereby exploring the morphological changes that have been involved in the evolution of bryophyte-feeding.

## Materials and Methods

### Sampling and Preparation

Eggs and larvae of bryophyte-feeding rhagionids were obtained from liverwort and moss mats infested with rhagionid flies from four locations in Japan: Kibune, Kyoto Prefecture; Akame Falls, Mie Prefecture; Nanataki, Wakayama Prefecture; Akiyamago, Niigata Prefecture; Nukabira, Hokkaido. No specific permission was required for the sampling, because the sampling sites were located outside of protected areas of national forests. Also, the insects and bryophytes sampled for this study are neither endangered nor protected. For rearing the larvae, plant materials were packed in small plastic cases. The cases were occasionally moistened and were maintained at room temperature (15 ± 5°C). First instar and/or mature larvae of each species were fixed and stored in 70% ethanol for morphological study. The collected larvae of *Litoleptis* were identified to species based on adult morphology. However, it was not possible to distinguish the species of *Ptiolina* and *Spania* due to several undescribed species existing in Japan (Imada, Y & Kato, M, *pers*. *obs*.).

### Morphology

To examine the external morphology of the larvae, ethanol-fixed specimens were dehydrated in absolute ethanol, submersed in Hexamethyldisilazane (HMDS), air-dried, and examined by a scanning electron microscope (SEM, Real Surface View Microscope VE-9800, Keyence Corp.) at 1–2 kV. To examine the mouthparts and associated inner structures that were not externally visible, head capsules were prepared by immersing them in hot KOH for 20 min, rinsed with distilled water, and then preserved in glycerin. The head capsules were dissected carefully under a microscope (StereoZoom Leica S8 APO, Leika Microsystems), and observed under an optical microscope (CX 40, Olympus). The terminology for morphological features generally follows that of [2], and [4] for mandibular terms. All of the examined materials in this study are deposited at the Graduate School of Human and Environmental Studies, Kyoto University, Kyoto, Japan (KUHE).

### Adult Oviposition Behavior

Although adults of bryophyte-feeding rhagionids were rarely found in the field, female oviposition behavior of 13 females from three species were observed: each one of *Spania* sp. (n = 5), *Lioleptis japonica* Imada & Kato (n = 4), *L*. *kiiensis* (n = 1), and *Ptiolina* sp. (n = 3) in May and June 2015 and in May 2016. The female behaviors were recorded on video (S2–S4 Files). After the sequence of female behaviors had finished, the oviposited eggs were collected with plant materials and maintained under laboratory conditions. Later, the adults of *Spania* and *Litoleptis* were collected to identify the species, but no *Ptiolina* was collected.

### Laboratory Methods for Molecular Data

Genomic DNA was extracted from thoracic muscle tissue preserved in absolute ethanol with the DNeasy Tissue Kit (Qiagen, Valencia, CA). Primer sets for the PCR and cycle sequencing (CS) reactions were completed using rc28C [31] and 28S-Dipt-7176R [32] for PCR and CS, and internal primers (rc28C, rc28P, rc28D, rc28Q, rc28H, 28C, 28E, 28H, 28K, 28P, 28Z)[31] for CS. PCR and DNA sequencing were performed using a modified method described in [18]. PCR was performed using a Biometra PCR machine with the following program: 95°C initial denature step of 3 min, followed by an amplification cycle of 95°C for 20 s, 54°C for 20 s, and 75°C for 1 min and 10 s. The cycle was repeated 30 times. After 10 min at 75°C, the products were cooled to 4°C. Sequence fragments were edited and compiled using the computer program MEGA 6 [33]. The aligned molecular dataset consisted of 2813 bp of 28S rRNA gene fragments. The sequence data were then aligned manually using the 28S rRNA alignment data published in [18] as a reference, and deposited at TreeBASE (http://www.treebase.org). All voucher specimens are preserved at the Graduate School of Human and Environmental Studies, Kyoto University, Japan (GHES). Newly obtained sequences are deposited at the International Nucleotide Sequence Database Collaboration (INSDC) through the DNA Data Bank of Japan (DDBJ).

### Dataset and Phylogenetic Analysis

A phylogenetic relationship of Spaniinae was inferred based on 28S rRNA sequences. The data set consisted of 8 outgroup and 12 ingroup taxa, of which 13 sequences were produced by [18] and six sequences were newly obtained for this study (Table 1). Phylogenetic trees were constructed using maximum likelihood (ML) and Bayesian inference (BI). ML phylogenies were constructed using RAxML v. 8.2.10 [34] with the GTR Gamma model. Branch support values were computed via 1000 non-parametric bootstrap replicates. Bootstrap bipartitions were drawn on the best tree topology. BI and Bayesian posterior probabilities (BPPs) were estimated using MrBayes v. 3.2.5 [35]. The GTR+I+G model was selected as the best-t partition scheme and models, based on the Bayesian information criterion [36] using Kakusan4 [37]. Two independent runs of four Markov chains were conducted for 20 million generations, and the tree was sampled every 100 generations. The parameter estimates and convergence were checked using Tracer v. 1.6.0 [38], and the rest of the 10,001 trees were discarded based on the results. Posterior probabilities of less than 0.95 were considered low and only indicate weak support for nodes, whereas those greater than 0.95 strongly indicate monophyly.

**Table 1:** Samples used for the molecular phylogenetic analyses. The arrangement of subfamilies adopted here is based on [16]. Samples shown with asterisk (*) were used as the outgroup taxa.

Subfamily	Species	Accession number of DDBJ or GenBank	Source	Locality
Arthrocerinae	*Arthroceras fulvicorne* Nagatomi, 1966*	DQ415517	Genbank	USA: UT
Bolbomyiidae	*Bolbomyia nana* Loew, 1862*	DQ415526	Genbank	Quebec
Chrisopilinae	*Chrysopilus quadratus* (Say, 1823) *	DQ415527	Genbank	USA: MD
Chrisopilinae	*Chrysopilus rhagiodes* Bromley & Curran, 1931*	DQ415528	Genbank	Costa Rica
Rhagioninae	*Arthroteles cinerea* Stuckenberg, 1956*	HM770491	Genbank	South Africa
Rhagioninae	*Atherimorpha montana* Hardy, 1927*	DQ415522	Genbank	Tasmania
Rhagioninae	*Rhagio hirtus* Loew, 1861* Rhagioninae	DQ415535	Genbank	USA: IL
Rhagioninae	*Rhagio mystaceus* (Macquart, 1840) *	DQ415536	Genbank	USA: IL
Spaniinae	*Litoleptis japonica* Imada & Kato, 2016	LC149449	New Sequence	Japan: Kyoto
Spaniinae	*Litoleptis kiiensis* Imada & Kato, 2016	LC149450	New Sequence	Japan: Wakayama
Spaniinae	*Omphalophora fasciata* (Loew, 1869)	DQ415534	Genbank	Saskatchewan
Spaniinae	*Ptiolina* sp.	HM770492	Genbank	USA: CO
Spaniinae	*Ptiolina* sp. Rh0373	LC149448	New Sequence	Japan: Iwate
Spaniinae	*Spania* sp. Rh0371	LC149451	New Sequence	Japan: Kyoto
Spaniinae	*Spania* sp. Rh0372	LC149452	New Sequence	Japan: Fukuoka
Spaniinae	*Spaniopsis clelandi* Ferguson, 1915	DQ415537	Genbank	Tasmania
Spaniinae	*Spaniopsis longicornis* Ferguson, 1915	DQ415538	Genbank	Australia
Spaniinae	*Symphoromyia atripes* Bigot, 1887	AF238559, AF238535	Genbank	USA: IL
Spaniinae	*Symphoromyia crassicornis* (Panzer, 1806)	LC149447	New Sequence	Japan: Nagano
Spaniinae	*Symphoromyia hirta* Johnson, 1897	DQ415539	Genbank	USA: IL

## Results

### Descriptions of Immature Stages

**Fig 1:**
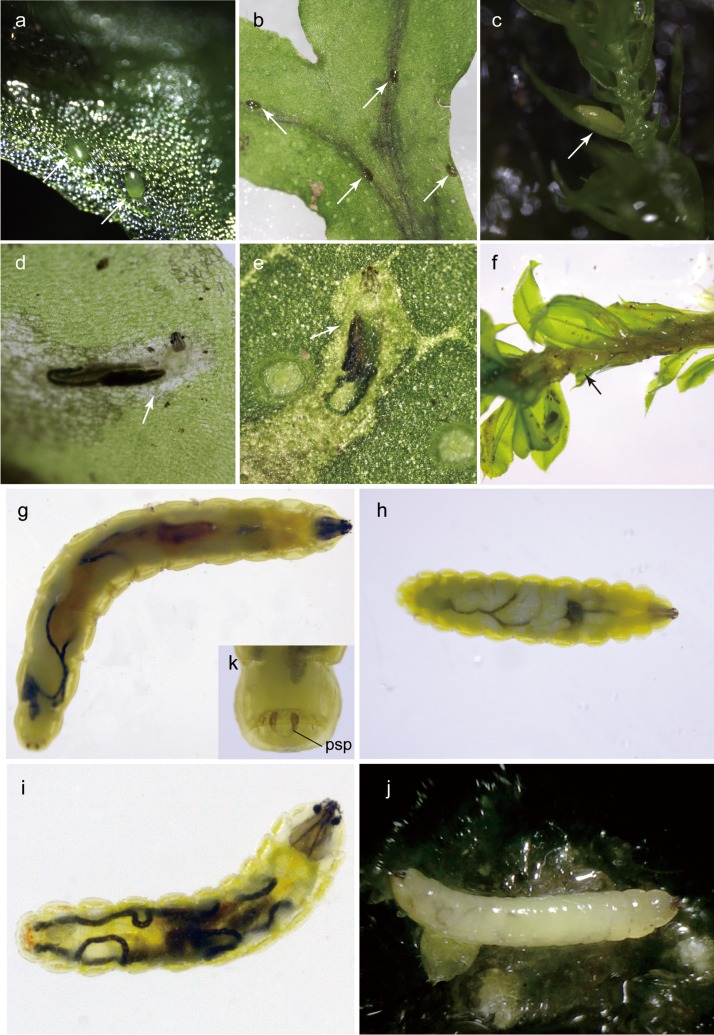
Eggs and larvae of four species of bryophyte-feeding Rhagionidae. Eggs (a-c): (a) eggs of *Spania* sp. (arrows), deposited on *Pellia endiviifolia*; (b) eggs of *Litoleptis japonica* (arrows), deposited on *Conocephalum conicum;* (c) eggs of *Ptiolina* sp. (arrows), deposited on *Brachythecium buchananii*. Larvae from life (d-f): (d) first instar larva of *Spania* sp., mining in a thallus of *P*. *endiviifolia*; (e) first instar larva of *Litoleptis japonica*, mining in a thallus of *C*. *conicum*; (f) second instar larva of *Ptiolina* sp. (arrow), boring a shoot of *Plagiomnium vesicatum*. Final instar larvae (g-j): (g) larva of *Spania* sp.; (h) larva of *Litoleptis japonica*; (i) larva of *L*. *kiiensis*; (j) larva of *Ptiolina* sp. (k) posterior spiracle (psp) of the final instar larva of *Spania* sp.

**Fig 2:**
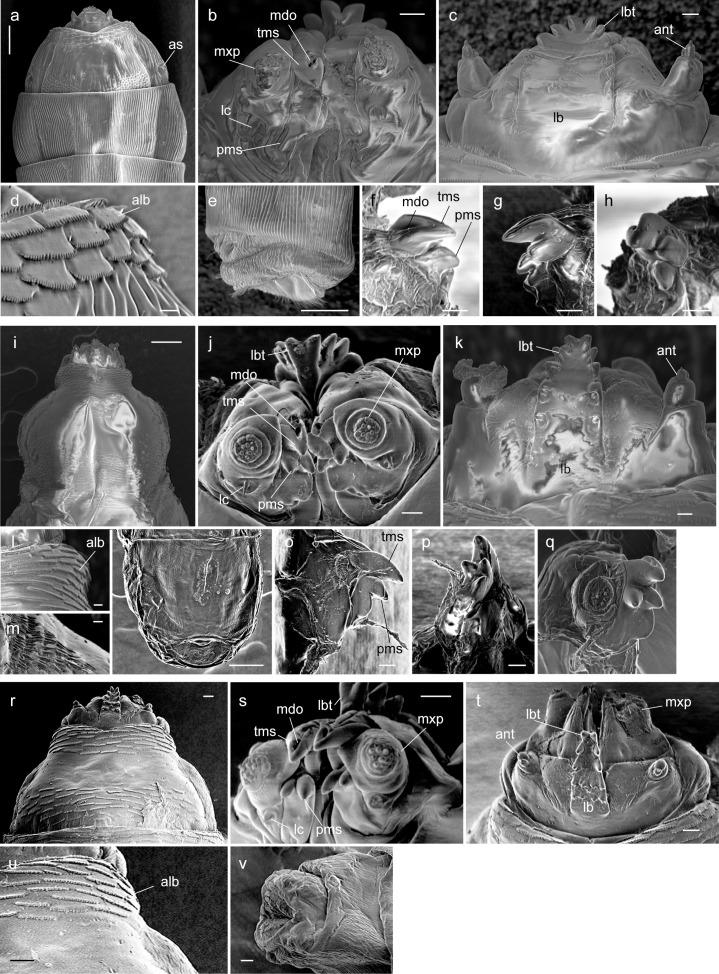
Larvae of three rhagionid species, SEM views. *Spania* sp. (a-h): (a) anterior body part, dorsal view (100 μm); (b) head, ventral view; (c) ditto, dorsal view; (d) anterior edge of first thoracic segment, dorsal view; (e) last abdominal segment, lateral view (100 μm); (f) right mandible, outer view; (g) ditto, inner view; (h) ditto, inner oblique view. *Litoleptis japonica* (i-q): (i) head and first thoracic segment, dorsal view (100 μm); (j) head, ventral view; (k) ditto, dorsal view; (l) anterior edge of first thoracic segment, dorsal view; (m) microtrichea at lateral side of first thoracic segment; (n) last abdominal segment, dorsal view (100 μm); (o) ditto, lateral view; (p) ditto, oblique ventral view; (q) ditto, oblique outer view. *L*. *kiiensis* (r-v): (r) head and first thoracic segment, dorsal view; (s) head, ventral view; (t) head, dorsal view; (u) anterior edge of first thoracic segment, dorsal view; (v) last abdominal segment, lateral view. Scale lengths in parentheses. Scale lengths 10 μm, where not shown. *Abbreviations*: *alb = anterior lobes of cuticle; ant = antenna; lbr = labrum; lbt = labral teeth; lc = lacinia; mdo = mandibular orifice; mxp = maxillary palp; pms = lower projections of apical mandibular sclerite; tms = upper tooth of apical mandibular sclerite*.

**Fig 3:**
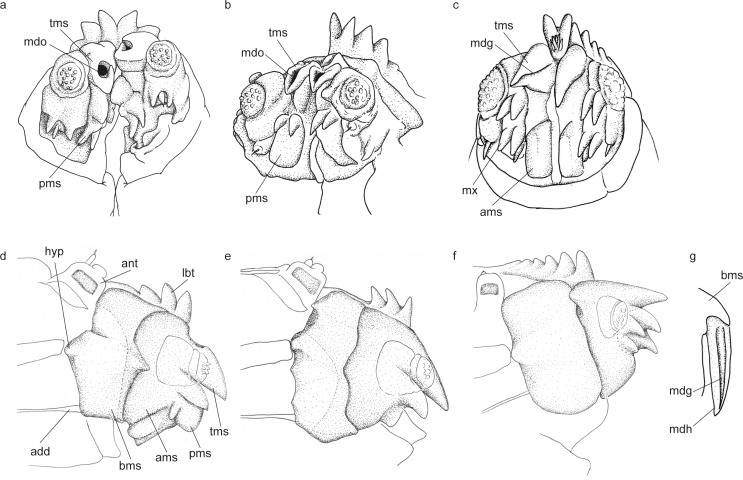
Cephalic features of final instar larvae of bryophyte-feeding rhagionids. Cephalic features illustrated based on SEM view, ventro-frontal view (a-c): (a) *Spania* sp.; (b) *Litoleptis japonica*; (c) *Ptiolina* sp. Cephalic skeleton features illustrated based on the dissected specimens, aboral view (d-f): (d) *Spania* sp.; (e) *Litoleptis japonica*; (f) *Ptiolina* sp. (g) mandibular hook of *Symphoromyia* sp., illustrated based on [17]. *Abbreviations*: *add = adductor apodeme; ant = antenna; ams = apical mandibular sclerite; bms = basal mandibular sclerite; hyp = hypocondyle; lbt = labral teeth; mdg = mandibular groove; mdo = mandibular orifice; mdh = mandibular hook; mx = maxilla; pms = lower projections of apical mandibular sclerite; tms = upper tooth of apical mandibular sclerite*.

*Spania* sp. (Figs 1A, 1D, 1G, 2A–2H, 3A and 3D)

#### Eggs

(Fig 1A) Length 0.42 mm long, flattened, oval, and entire surface smooth. White and glossy when first deposited, but became pale yellow a few days after oviposition.

#### Larvae

*First instar larva* (Fig 1D): length 0.5–0.8 mm long, pale yellow. *Final instar larva* (Fig 2A–2H): length 4.0–5.5 mm long, pale yellow. Body with 11 segments. Antenna (Fig 2C) two-segmented, cylindrical, short. Eyes (stemmata) conspicuous on anterolateral margin of tentorial phragma. First thoracic segment (Fig 2A) gradually broadens toward posterior end. Body segments covered with longitudinal wrinkles, creeping welts absent. Thoracic segments with transverse rows of scale-like lobes, with jagged posterior margins (Fig 2D) at anterior edge of first thoracic segment and segmental boundaries of all thoracic segment for both dorsal and ventral surfaces. Head capsule 0.5 mm long, dark brown, dorsoventrally flattened, longitudinally extended trapezoid, only partly protruding into second segment (Fig 1G). Labrum (Fig 2C) well developed, well sclerotized, width at base wider than half distance between bases of antennae. Labral teeth (Fig 2C) heavily sclerotized, five pairs in two rows, converging anteriorly, with labral papillae in posterior two pairs. Mandible consists of two articulated sclerites: basal mandibular sclerite and apical mandibular sclerite (Fig 3D). Apical mandibular sclerite (Figs 2B, 2F–2H, 3A and 3D) consists of one large upper tooth, and two projections with blunt apex underneath; upper tooth flat dorsally, with wide dorsal orifice (Fig 2B and 2F) interiorly forming channel. Maxilla (Figs 2B and 3A) completely fused to basal mandibular sclerite, more or less membranous. Maxillary palpus (Fig 2B) soft and three-segmented, with lacinia (Fig 2B) underneath. Labium strongly reduced or absent. Last abdominal segment (Fig 2E) with shallow emargination, without lobes. Amphipneustic: anterior spiracles (Fig 2A) at posterolateral margin of second segment; posterior spiracles (Fig 1K) oval, situated at base of dorsal side of emargination.

#### Materials examined

Japan, Kyoto: 10 individuals, Kibune, 18-XII-2015, Y. Imada.

#### Diagnosis

Final instar larvae of *Spania* sp. may be immediately distinguished from other genera of Rhagionidae by the longitudinal wrinkles or striations of the cuticle of the thorax and abdomen. As well as in *Litoleptis*, creeping welts are absent and the apical mandibular sclerite is composed of a tooth and two teeth-like projections underneath. The mandible of this species lack the adoral groove. Instead, the mandibular orifice is present on the dorsal surface leading to internal canal, which is also present in *Litoleptis* spp.

#### Biology

Eggs and larvae of *Spania* sp. were exclusively found on the thallose liverwort, *Pellia endiviifolia* (Dicks.) Dumort. (Marchantiophyta: Pelliaceae) in the field. One to six eggs were laid near the edge of each thallus (Fig 1A). Oviposited eggs adhered to the adaxial side of the thallus of the host-plant. Early stage mines were linear, but became marked with blotches as the larvae grew. Mines generally began along the edge of thalli, and extended near the costa (mid-vein); however, no clear mining pattern was detected. Larvae were sometimes found outside of the thalli. Larvae also had the ability to leave their primary mines and bore secondary mines in another thallus of their host-plant, which was confirmed in the laboratory. This migratory habit was observed when the mine and/or food conditions became adverse in the initial thallus. For final instars, all mines were found to occur on a single thallus, and the larvae were inactive. Full-grown larvae appeared to be in the third instar. Larvae underwent pupation inside their mine during early spring. Adults emerged in spring (March–May).

*Litoleptis japonica* Imada & Kato, 2016 (Figs 1B, 1E, 1H, 2I–2Q, 3B and 3E)

#### Egg

(Fig 1B) Length 0.4 mm long, flattened, oval, and entire surface smooth. White and glossy when first deposited, but became pale yellow a few days after oviposition.

#### Larva

*First instar larva* (Fig 1E): length 0.5–0.8 mm long, pale yellow. *Final instar larva* (Fig 2I–2Q): length 3.7–4.5 mm, pale yellow. Body with 11 segments. Antenna (Fig 2K) two-segmented, cylindrical, short. Eyes (stemmata) conspicuous on anterolateral margin of tentorial phragma. First thoracic segment (Fig 2I) narrowest at anterior end, approximately half as wide as widest part. Body segment surface smooth, but bear microtricheae on anterolateral surface of first thoracic segment (Fig 2M); creeping welts absent. Thoracic segments with scale-like lobes shallowly laciniated along posterior margin (Fig 2L), at anterior and posterior edge of first segment thoracic segment and segmental boundaries of all thoracic segments on both ventral and dorsal surfaces. Head capsule 0.8 mm, dark brown, dorsoventrally flattened, longitudinally extended trapezoid, only partly protruding into second segment (Fig 1H). Labrum (Fig 2K) well developed, well sclerotized, width at base more or less 1/3 of distance between bases of antennae. Labral teeth (Fig 2J and 2K) heavily sclerotized, five pairs in two rows, converging anteriorly, with labral papillae on posterior two pairs. Mandible consists of two articulated sclerites: basal mandibular sclerite and apical mandibular sclerite (Fig 3E). Apical mandibular sclerite (Figs 2J, 2O–2Q, 3B and 3E) consists of two components: one strongly developed upper tooth, closely attached to a pair of extended projections underneath; upper tooth flat dorsally, with orifice (Figs 2J and 3B) dorsomedially, leading to channel interiorly. Maxilla completely fused to basal mandibular sclerite, more or less membranous. Maxillary palpus (Fig 2J) soft and three-segmented, with spine-like lacinia (Fig 2J) underneath. Labium strongly reduced or absent. Last abdominal segment (Fig 2N) with shallow emargination, without lobes. Metapneustic; posterior spiracles oval, situated at base of dorsal side of emargination.

#### Materials examined

Japan: Kyoto: 10 individuals, Kibune, 18-XII-2015, Y. Imada; Mie: 2 individuals, Akame Falls, 16-I-2016, Y. Imada; Wakayama: 2, Nanataki, 14-IX-2015, Y. Imada.

#### Diagnosis

Final instar larvae of *Litoleptis japonica* resembles *Spania* in general appearance, but may be distinguished based on the absence of the longitudinal wrinkles or striations of the cuticle of the thorax and abdomen. *L*. *japonica* resembles *L*. *kiiensis*, and may be distinguished by the following characteristics: body size > 3.5 mm (as opposed to <1.5 mm in *L*. *kiiensis*); two lower projections of apical mandibular sclerite are attached to each other (but are detached in *L*. *kiiensis*).

#### Biology

Eggs and larvae of *L*. *japonica* were exclusively found on the thallose liverwort, *Conocephalum conicum* (L.) Dum. (Marchantiophyta: Conocephalaceae) in the field (n = 72). One to six eggs were laid per thallus, near the edge of abaxial surface (Fig 1B). Oviposited eggs adhered to the adaxial side of the thallus of the host-plant. Early stage mines were linear, but became marked with blotches as the larvae grew. Mines generally began near the costa (mid-vein) and extended along it; however, the mine shapes varied considerably, with no constant mining pattern. Larvae had the ability to leave their primary mine and bore a secondary mine in another thallus of their host-plant. This migration behavior was observed once in the field. In the final instar, mines were singly found on a thallus. Larvae underwent pupation inside their mine during early spring. Full-grown larvae appeared to be third instars. Adults emerged in spring (March–May).

*Litoleptis kiiensis* Imada & Kato, 2016 (Figs 1I, 2R–2V)

#### Egg

Unknown.

#### Larva

*Final instar larva* (Fig 2R–2V): length 1.0–1.5 mm, pale yellow. Body with 11 segments. Antenna (Fig 2T) two-segmented, cylindrical, short. Eyes (stemmata) conspicuous on anterolateral margin of tentorial phragma. Body segment surface smooth and glabrous; creeping welts absent. Thoracic segments with transverse rows of scale-like lobes laciniated along posterior margin (Fig 2U), at anterior and posterior edge of all thoracic segments, both on ventral and dorsal margins. Head capsule 0.2 mm, dark brown, dorsoventrally flattened, longitudinally extended trapezoid, only partly protruding into second segment (Fig 1I). Labrum (Fig 2T) well developed, well sclerotized, width at base more or less 1/3 of distance between bases of antennae. Labral teeth (Fig 2S and 2T) heavily sclerotized, five pairs in two rows, converging anteriorly, with labial papillae on posterior two pairs. Mandible consists of two articulated sclerites: basal mandibular sclerite and apical mandibular sclerite. Apical mandibular sclerite (Fig 2S) consists of one comparatively large tooth and a pair of extended projections underneath; upper tooth flat dorsally, with orifice (Fig 2S) dorsomedially, leading to channel interiorly. Maxilla completely fused to basal mandibular sclerite, more or less membranous. Maxillary palpus (Fig 2S) soft and three-segmented, with spine-like lacinia (Fig 2S) underneath. Labium strongly reduced or absent. Last abdominal segment (Fig 2V) with shallow emargination, without lobe. Metapneustic; posterior spiracles oval, situated at base of dorsal side of emargination.

#### Materials examined

Japan: Wakayama: 4 individuals, Nanataki, 14-IX-2015, Y. Imada.

#### Diagnosis

Final instar larvae of *L*. *kiiensis* resemble *L*. *japonica*, but may be distinguished by their body length (<2.0 mm, as opposed to >3.0 mm in *L*. *japonica*) and the absence of tufts of microtricheae on the anterolateral surface of the first thoracic segment.

#### Biology

Larvae of *L*. *kiiensis* were thallus-miners of *Reboulia hemisphaerica* (L.) Raddi (Marchantiophyta: Aytoniaceae). Larvae mined in the middle layer of the thalli. It was quite difficult to recognize active mines from the outside due to the thickness of the thalli of *R*. *hemisphaerica*. Final instar larvae singly mined along the costa (mid-vein), and pupated near the adaxial layer of the thalli inside their mine during early spring, which was visually recognizable from the outside. Adults emerged in spring (March–May, under laboratory conditions).

**Fig 4:**
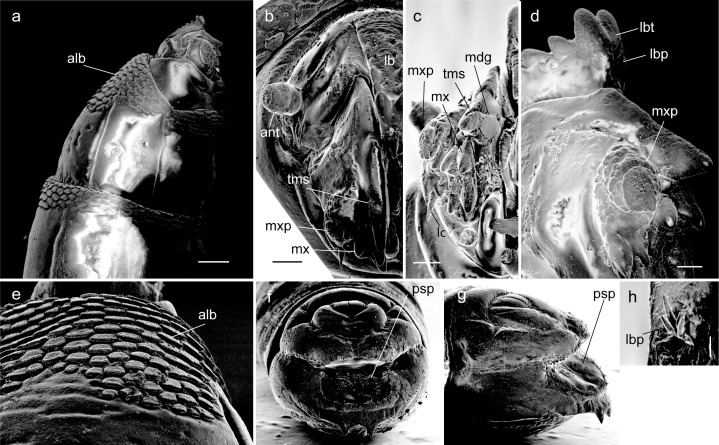
Larva of *Ptiolina* sp., SEM views. (a) Anterior part of body, lateral view (100 μm); (b) head, dorsofrontal view (30 μm); (c) anterior part of body, frontal view (30 μm); (d) anterior part of body, outer view (20 μm); (e) first thoracic segment, inner view (30 μm); (f) last abdominal segment, posteroventral view (30 μm); (g) last abdominal segment, oblique ventral view (30 μm). Scale lengths in parentheses. *Abbreviations*: *ant = antenna; alb = anterior lobes of cuticle; tms = upper tooth of apical mandibular sclerite; lb = labrum; lbp = labral papillae; lbt = labral teeth; lc = lacinia; mdg = mandibular groove; mx = maxilla; mxp = maxillary palp; psp = posterior spiracle*.

*Ptiolina* sp. (Figs 1C, 1F, 1J, 3C, 3F and 4A–4H)

#### Egg

(Fig 1C) Length 0.68 mm long, pale yellow, flattened, oval and entire surface smooth.

#### Larva

*Final instar larva* (Fig 4A–4H): length 6.3 mm, white to pale yellow. Body with 11 segments. Antenna (Fig 4B) one-segmented, cylindrical, short. Eyes (stemmata) present on anterolateral margin of tentorial phragma. Body segment surfaces smooth and glabrous; creeping welts present. Thoracic segments with several rows of developed lobes deeply laciniate along entire margin (Fig 4A and 4E) at anterior and posterior edge of first segment, and segmental boundaries of all thoracic segments on both ventral and dorsal surfaces. Head capsule 1.0 mm long, dark brown, dorsoventrally flattened, longitudinally extended trapezoid, only partly protruding into second segment. Labrum (Fig 4B) well developed, well sclerotized, width at base narrower than 1/3 of distance between bases of antenna. Labral teeth (Fig 4D) heavily sclerotized, five pairs in two rows, converging anteriorly; labral papillae (Fig 4D and 4H) present in posterior two pairs of labral teeth and at front of labrum. Labial sclerite absent. Mandible consists of two sclerites: basal mandibular sclerite and apical mandibular sclerite (Fig 3F). Apical mandibular sclerite (Figs 3C, 3F, 4B and 4C) consists of blade-shaped upper tooth and four lower projections on its outer side; mandibular groove present on adoral surface of upper tooth (Fig 4C). Maxilla (Fig 4C) completely fused to basal mandibular sclerite, more or less membranous. Maxillary palpus (Fig 4B and 4C) soft, poorly differentiated, three-segmented; with spine-like lacinia (Fig 4C) underneath. Labium strongly reduced or absent. Last abdominal segment (Fig 4F and 4G) large, with two lobes; ventral lobe with shallow emargination along outer margin: dorsal lobe with two projections oriented upwards (Fig 4G). Amphipneustic: anterior spiracles at posterolateral margins of second segment; posterior spiracles (Fig 4F and 4G) oval, situated at base of dorsal lobe.

#### Material examined

Japan: Hokkaido: 1 individual, Nukabira, 7-VII-2016, Y. Imada; Niigata: 1 individual, Akiyamago, 3-V-2015, M. Kato.

#### Diagnosis

The final instar larvae of *Ptiolina* sp. are distinguished from other genera of Rhagionidae based on the presence of labral papillae at the front of the labrum. This species shares well-developed maxillary teeth with the species described by [16], but differs from it in the number of sclerotized teeth in the maxilla (versus three). Mandibular groove is present on the adoral surface of the apical mandibular sclerite, as well as in species of *Chrysopilus*, *Rhagio*, and *Symphoromyia*. Creeping welts are present, as opposed to absent in *Spania* and *Litoleptis*. Base of apical mandibular sclerite of this species protrudes ventrally (Fig 3F and 4C), as it is visible from outside of the body, whereas it is concealed for the species of *Spania* (Fig 2B and 3D) and *Litoleptis* (Fig 2J, 2S and 3E).

#### Biology

The following account is based on the unidentified species of *Ptiolina* found in Kibune, Kyoto Prefecture, Japan, which was reared at room temperature (15 ± 5°C). Eggs were deposited on separate thalli or leaves of *Brachythecium buchananii* (Hook.) Jaeg. (Bryophyta: Brachytheciaceae), *Pellia endiviifolia* (Dicks.) Dumort. (Marchantiophyta: Pelliaceae), and *Heteroscyphus coalitus* (Hook.) Schiffn. (Marchantiophyta: Geocalycaceae). Larvae hatched one week after oviposition. The larva then buried its head deep into the base of the shoot of *B*. *buchananii*. When the larva did not forage for the food-plant, it rested in the damp soil. A second instar larva of *Ptiolina* sp. was also found in Nukabira, Hokkaido, Japan, which was a stem borer of *Plagiomnium vesicatum* (Besch.) T.J.Kop (Bryophyta: Mniaceae). So far, adults of *Ptiolina* spp. emerged from the colonies of *Taxiphyllum aomoriense* (Besch.) Z. Iwats. (Bryophyta: Hypnaceae), *B*. *plumosum* (Hedw.) Schimp., and *Myuroclada maximowiczii* Steere & Schofield (Bryophyta: Brachytheciaceae), and thus these mosses are potential host-plants of *Ptiolina*. Adults emerged from April to June.

### Larval Feeding Behavior

The movement of mouthparts of the first instar larvae of *Spania* sp. was analyzed during feeding (S1 File). Larvae scraped plant tissues by concurrently moving their maxillo-mandibles vertically, and actively dug feeding tunnels. Afterwards, larvae imbibed fragmented plant tissues into the mouth for a few minutes, while keeping their mandibles adducted. During the suctorial feeding process, the fragmented plant tissues directly passed through the esophagus with large amounts of sap filling up the mine. The food was then transported into the digestive tract without being strained.

The feeding behavior of *Ptiolina* sp. at the second instar was observed (Fig 1F), although the operation of mouthparts remains uncertain, due to the concealed way of feeding. The larva usually rested below the shallow soil, when it did not feed. When the larva fed, the larva stuck its head from the stem base of *Plagiomnium vesicatum*. The larva then moved through the stem towards the apex of the shoot. The larva was a stem borer and did not feed on the leaves of the moss. After the larva reached the shoot apex, it slowly moved backward and rested below the soil near the base of the shoot.

### Adult Oviposition Behavior

*Spania* sp.

**Fig 5:**
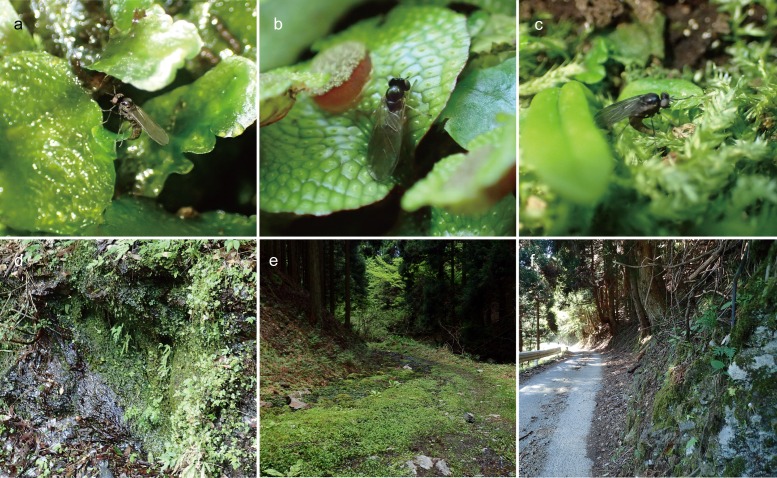
Ovipositing adult females and habitats of the bryophyte-feeders. Adult behaviors (a-c): (a) female of *Spania* sp. ovipositing on *Pellia endiviifolia*, at Higashiyoshinomura, Nara Pref., Japan; (b) female of *Litoleptis japonica* ovipositing on *Conocephalum conicum*, at Kibune, Kyoto Pref., Japan; (c) female of *Ptiolina* sp. ovipositing on *Brachythecium buchananii* at Kibune, Kyoto Pref., Japan. Habitats (d-f): (d) *Spania* sp.; (e) *L*. *japonica*; (f) *Ptiolina* sp.

An ovipositing female was observed on 1 May, 2015 at Higashiyoshino-mura, Nara Prefecture, Japan. The habitat was a moist valley with steep cliffs near a waterfall that was dominated by ferns and bryophytes. The female *Spania* was found on a tuft of liverwort growing on a rocky cliff moistened by trickling water, along a forest roadway though the deciduous forest (Fig 5D). The prominent bryophytes at this site were *Pellia envidiifolia*, *Conocephalum conicum* and *Dumortiera hirsuta* (Sw.) Nees. Adult flies emerged during the season when new thalli of the thallose liverworts emerged.

The sequence of oviposition behavior was observed for 70 min (1:30 p.m.–2:40 p.m.) and recorded on video (S2 File). Females perched on the thallus of *Pellia* or just walked around the tufts of *Pellia* liverwort when they did not oviposit. They usually did not disperse far from the tufts of *Pellia* liverwort. The female searched for thalli on which to oviposit, walking around the liverwort mats without flying. As she encountered a newly sprouted thallus of *Pellia*, she bent her stretched abdomen and deposited each egg separately on the abaxial surface of the thallus (Fig 5A). She often deposited more than one egg on the same thallus. After oviposition, she retracted her abdomen and started walking. She repeatedly performed this behavioral sequence, and infrequently hovered unsteadily 30–100 cm away from the colony of liverworts. When she landed at other sites on the plant, she resumed the sequence of oviposition behavior. Sometimes she flew over the plant, but flight was abrupt and short, and did not last more than 5 sec.

Although the liverworts and mosses were intermingled together at the observation site, the eggs (n = 25) were only laid on *P*. *endiviifolia* during the observation (Fig 5A). All thallose liverworts occurring at this site (i.e., *P*. *envidiifolia*, *C*. *conicum*, and *D*. *hirsuta*) were exhaustively examined after observation. Other eggs from the same species (n = 21) were also detected, but only on *P*. *endiviifolia*.

*Litoleptis* spp.

The ovipositing females of *Litoleptis japonica* were observed on 13 May 2016, at Kibune, Kyoto Prefecture, Japan. The observation site was a steep slope facing a forest path along a stream, with a deciduous forest that is dominated by *Aesculus turbinata* Blume (Sapindales: Hippocastanaceae) and *Cercidiphyllum japonicum* Siebold & Zucc. (Saxifragales: Cercidiphyllaceae) (Fig 5E). Four female *Litoleptis* were found on a tuft of liverwort growing on a clayey slope. *Conocephalum conicum* was dominant at this site.

The sequence of oviposition behaviors was observed for an hour (13:00 p.m.–14:00 p.m.) and recorded on video (S3 File), on 13 May 2016. While searching for thalli on which to oviposit, the female usually walked around the liverwort mats, and actively flied over the liverwort mats. As she encountered a thallus of *Conocephalum*, regardless of the age of thalli, she pressed her abdomen against the thallus and deposited each egg separately on the adaxial surface of the thallus (Fig 5B). She often deposited more than one egg on the same thallus. After oviposition, she retracted her abdomen and started walking. She repeatedly performed this behavioral sequence, and infrequently hovered unsteadily 30–100 cm away from the colony of liverworts. When she landed at other sites on the plant, she resumed the sequence of oviposition behavior.

In addition to the ovipositing females, the eggs of this species were collected during April and May at Kibune, Kyoto Prefecture and at Akame Falls, Mie Prefecture, Japan. Four species of thallose liverworts that are common at these sites, *C*. *conicum*, *Reboulia hemisphaerica*, *Pellia endiviifolia*, and *Makinoa crispata* (Steph.) Miyake (Marchantiophyta: Makinoaceae) were examined. All eggs of *L*. *japonica* were exclusively found on the adaxial surface of the thalli of their host-plant, *C*. *conicum* (n = 75).

The oviposition behavior of *L*. *kiiensis* Imada & Kato was not observed in the field; however, we succeeded in observing it in captivity. On 6 May, 2015, three females and one male emerged from the liverwort mats of *R*. *hemisphaerica*, which were collected from Uchinami in Fukui Prefecture, Japan and were kept in a plastic case. The observation was conducted for 13.5 hours (from 19:30 on 6 May, 2015, to 10:00 on 7 May, 2015). After walking around the liverwort mats, one female laid an egg on the adaxial surface near the costa of a thallus of *R*. *hemisphaerica*, perching in the middle of the thallus.

*Ptiolina* sp.

The ovipositing behavior of *Ptiolina* sp. was observed three times on 22 and 25 May and June 2, 2015, at Kibune, Kyoto Prefecture, Japan. These behaviors were recorded on video (S4 File). The females could not be identified, since we failed to collect them after the observation. However, it may be *Ptiolina sphaeralis* Nagatomi, because this is the only *Ptiolina* species ever collected at the site (Imada Y, *pers*. *obs*.). The observation site was near the place where the females of *Litoleptis japonica* were observed. In the first observation, a female was found on a tuft of moss, *Brachythecium buchananii*, on a rocky cliff (Fig 5F). In the other cases, the females were found on a mixture of mosses and liverworts on the forest floor.

Each time, oviposition occurred between 15:00 and 17:00. The oviposition behavior was essentially similar in all three instances. When the female did not oviposit, she continued walking and occasionally fly amongst the mosses in shady places. The female walked swiftly on the bryophyte mats without flying, and intermittently deposited her eggs on them by randomly inserting her abdomen into the tufts of mosses and liverworts (Fig 5C), regardless of the bryophyte species. The female did not lay eggs multiple times on the same shoot. After oviposition, the female started walking, searching for another place to oviposit. While repeatedly performing the sequence of behavior, she occasionally hovered at a distance from the patch (ca. 3 m). On returning to the same patch, she resumed the sequence of oviposition behavior. At dusk, the female flied away from the oviposition site.

The first female laid all of her eggs on *B*. *buchananii* (n = 6), whereas the other females oviposited on three liverwort and moss species that were predominant at the site, *Pellia endiviifolia* (n = 1), *Heteroscyphus coalitus* (n = 1), and *B*. *buchananii* (n = 3).

### Molecular Phylogeny of Spaniinae

**Fig 6:**
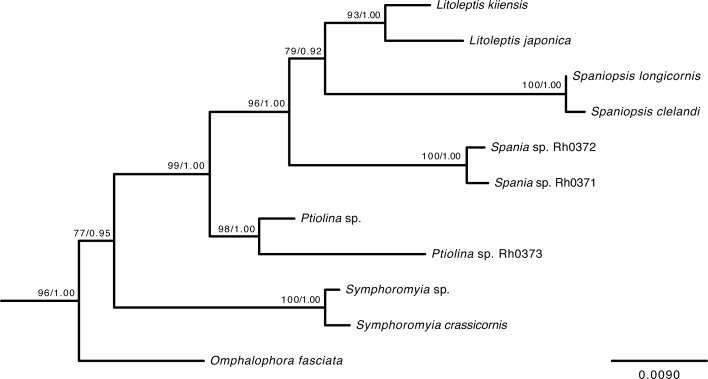
Bayesian inference tree of Spaniinae based on nuclear 28S rRNA. The maximum likelihood approach yielded the same topology. Numbers on nodes represent the bootstrap (BP) values for maximum likelihood and Bayesian posterior probabilities (BPP).

The BI tree of Rhagionidae based on 28s rRNA had an identical topology to that of the ML tree, and the resultant phylogeny of Rhagionidae was generally congruent with that of [18]. Also, the trees recovered the monophyly of Spaniinae (Fig 6), including the newly obtained sequences of *Litoleptis* and *Spania* (BS = 96%, BPP = 1.00). *Litoleptis* formed a monophyletic clade with *Spaniopsis* (BS = 79%, BPP = 0.92), although there was not sufficient BS and BPP support. This clade is a sister lineage to *Spania* (BS = 96%, BPP = 1.00), followed by *Ptiolina* (BS = 99%, BPP = 1.00), *Symphoromyia* (BS = 77%, BPP = 0.95), and *Omphalophora* (BS = 96%, BPP = 1.0) in this order.

## Discussion

### Life Histories of Herbivorous Rhagionidae

There are three genera of bryophyte-feeding rhagionids: *Spania*, *Litoleptis*, and *Ptiolina*. *Spania* and *Litoleptis* are thallus-miners, while *Ptiolina* is a stem borer of mosses. *Spania nigra* Meigen was previously reported to be a thallus-miner of *Pellia neesiana* (Gottsche) Limpr. (Marchantiophyta: Pelliaceae) [10, 12, 13], and used the same host-plant genus as *Spania* sp. examined in this study. Some *Ptiolina* species were previously reported to be moss feeders [10, 14, 28]; however, *Ptiolina obscura* Fallén may also feed on the liverwort, *Marchantia polymorpha* L. (Marchantiophyta: Marchantiaceae) [13]. Overall, the host-plant range of *Spania* and *Litoleptis* may be restricted to several specific liverwort families. Specifically, *Spania* is associated with Pelliaceae, and *Litoleptis* is associated with either Conocephalaceae or Aytonaceae. In comparison, *Ptiolina* feeds on Mniaceae and Brachytheciaceae, and possibly associated with Hypnaceae, Pelliaceae, and Geocalyceae.

Our observations on adult oviposition behavior indicate that the adult females of *Spania* and *Litoleptis* lay their eggs on the host-plants of their larvae. However, these genera exhibit different oviposition habits. Adult females of *Spania* lay eggs on the ventral surface of their host-plant by bending her abdomen. Generally, this oviposition behavior is achieved by retracting the terminal segments of telescopic abdomen, which seems to be an adaptation for oviposition in a terrestrial habitat [18]. By contrast, the females of *L*. *japonica* oviposit on the dorsal surface of their host-plant without bending her abdomen. One of the possible reasons underlying this unusual behavior may be the sporophyte morphology of their host-plants. Complex thalloid liverworts (e.g. Conocephalaceae and Aytoniaceae, the host-plants of *Litoleptis*) are internally differenciated into an upper layer of air chambers and a lower parenchymatous zone (i.e. thin-walled cells); however, simple thallose liverworts (e.g. Pelliaceae, the host-plants of *Spania*) lack such extensive differentiation [39]. Thus, the oviposition behavior of *Litoleptis* may be related to the characteristics of their host-plants, although the relationship is unclear.

### Putative Function of Larval Mouthparts of Thallus-Miners

According to the feeding behavior of the 1st instar larvae of *Spania* sp., the feeding behavior of the thallus-miners is assumed to consist of mastication and suction processes. First, the larvae scrape the ambient plant tissues. The apical mandibular sclerite of the thallus-miners, which is short with several teeth, may facilitate the effective grinding of plant tissues during the mastication. Second, in the suction process, the thallus-miners rapidly imbibe fragmented thallus tissues together with a large quantity of cell sap that fills the mine, with the mandibles being kept adducting. The mandibular orifice may play a major role in the suction feeding process, because the wide dorsal orifice forms an inward channel appearing to lead to the mouth. Despite the dorsal orifice of the thallus-miners being a novel character, it may have the similar function as the medial groove of Tabanomorpha.

In general, the mandibular groove on the adoral surface in the lower Tabanomorpha facilitates the flushing of liquid food into a food canal during feeding [4, 25, 40, 41]. For most lower Tabanomorpha, the two sides of the adoral groove of the apical mandibular sclerite must match so that the mandibles, as a whole, form a food canal when the hooks are appressed together during feeding [4, 25, 40, 41]. However, it has been suggested that a mandible with a medial groove is assumed to be the primitive state for Brachycera [18], based on the fact that two sides of the maxillo-mandibles do not necessarily operate coordinately, in many taxa of Tabanomorpha and Xylophagomorpha [39, 41]. Although the mandibular orifice of the thallus-mining genera appears to facilitate flushing the fragmented plant materials, its position and feeding process are considerably different from the other Tabanomorpha. In the thallus-miners, the two sides of the mandibles synchronously operate during feeding; however, the mandibular orifices do not join together due to their dorsal position.

### Morphological Changes in the Larvae of Bryophyte-Feeding Rhagionids

Our phylogeny provides a key to understand the evolutionary process of diet shifts leading to the bryophyte-feeding in Rhagionidae. The bryophyte-feeding genera were confidently placed within Spaniinae by the molecular phylogeny (Fig 6). All of the larvae of the bryophyte-feeders share a special cuticle structure at the anterior edge of the first thoracic segment (Figs 2D, 2L, 2U and 4E) and the similar structure has also been found in *Symphoromyia* and *Ptiolina* [18]. This result strengthens a prediction by [18] that suggest the scale-like anterior lobes of the first thoracic segment represent a synapomorphy of Spaniinae. Among known larvae of spaniine genera, the larvae of the thallus-mining genera, *Spania* and *Litoleptis*, morphologically resemble each other. It is correlated with the fact that *Litoleptis* is most closely related to *Spania* in terms of the female genital morphology [15]. However, these morphological predictions contradict the molecular phylogeny: the 28S rRNA tree placed *Spaniopsis* as the closest relative of *Litoleptis* within Spaniinae. *Spaniopsis* is a genus endemic to Australia, with the adult female being distinctive in that it has a blood-feeding habit; its larvae have yet to be examined. Importantly, *Symphoromyia*, of which larvae are presumably detritivores [26], was sister to a clade constituted by the bryophyte-feeding genera and *Spaniopsis*.

This study demonstrates several morphological modifications that have taken place during the evolution of bryophyte-feeding. In particular, it is shown that two characters widely occurring in Tabanomorpha are secondarily lost during the evolution of the bryophyte-feeding. Firstly, mandibular brush is absent in the thallus-miners, although it is a synapomorphic trait of Tabanomorpha [42, 43]. The mandibular brush is absent in the *Ptiolina* species examined in this study as well, and is reduced or remnant in the species observed by [18]. It is thought that the mandibular brush is used for anchoring the head capsule within prey in the carnivores [2]. The larvae of *Symphoromyia* also bear the mandibular brush jointed with simple fold of cuticle [18], although the function is unknown. Secondly, the thallus-mining genera do not possess creeping welts, while the other rhagionid genera including *Ptiolina* and *Symphoromyia* have the ventral creeping welts [18]. The absence of creeping welts in the thallus-mining genera is likely related to the endophagous feeding habit, because the ventral creeping welts are, in general, used for locomotion in terrestrial habitats [44]. These morphological features in the bryophyte-feeders are considered to be correlated to the specialized habitat and feeding modes.

The most notable feature of the bryophyte-feeding genera is the form of the larval mandibles and associated mouthparts. The prototype structure of the mandibles of Brachycera larvae, along with most Rhagionidae, is a long and apically blade-shaped mandibular hook. Even though *Symphoromyia* are not predacious, they have the hooked mandibles with adoral grooves as well as the predacious members of Rhagionidae (Fig 3G, see also [18]). By contrast, the mandibles of the thallus-mining genera are greatly reduced in size and are dorsally expanded. The maxillo-mandible of *Spania* and *Litoleptis* is primarily characterized by heavily sclerotized projections that lay beneath the upper tooth of the apical mandibular sclerite (Figs 2B, 2J, 2S, 3A and 3B). It is uncertain whether these projections are mandibles or fusion products of maxilla and mandible. *Ptiolina* has similarly developed projections; however, these projections are located on the outer side of the upper tooth of the apical mandibular sclerite and seem to pertain to the maxilla (Figs 3C and 4C). Furthermore, the thallus-miners share the mandibular orifice on the dorsal side of the apical mandibular sclerite (Figs 2B, 2J, 2S, 3A and 3B), whereas the mandibular groove of other Brachycera taxa occurs on the adoral surface of the mandibular hook (Fig 3G). In *Ptiolina*, a weak groove was present on the adoral surface of the mandibular hook (Fig 4C), although it appears not to retain its function. Due to the different position and shape of the mandibular orifice of the thallus-mining genera, it is likely not to be homologous to the adoral groove of the other genera of Rhagionidae.

Taken together, these findings suggest that the evolution of herbivory in Spaniinae is accompanied by the loss (or reduction) of the mandibular brush and adoral mandibular groove. Subsequently, the spaniines have achieved remarkable changes in a lineage of thallus-miners: the occurrence of toothed mandibles with dorsal orifice and the loss of creeping welts. These morphological changes may be led by their endophagous feeding habit and the surroundings filled with the plant fluids. Even among a wide variety of leaf-mining insects, suction feeding with the mandibular orifice may be a novel method of feeding. Further morphological examination of larvae with unusual feeding habits may reveal the morphological predispositions that have been involved in the frequent diet shifts in the evolutionary history of Diptera.

## Supporting Information

S1 File Larval feeding behavior of *Spania* sp. at the 1st instar, ventral view. (MP4)

S2 File Adult female oviposition behavior of *Spania* sp. (MP4)

S3 File Adult female oviposition behavior of *Litoleptis japonica*. (MP4)

S4 File Adult female oviposition behavior of *Ptiolina* sp. (MP4)

## References

[1] Wiegmann BM, Trautwein MD, Winkler IS, Barr NB, Kim J-W, Lambkin C, et al Episodic radiations in the fly tree of life. Proc Natl Acad Sci USA 2011;108: 5690–5695. 10.1073/pnas.1012675108 21402926 PMC3078341

[2] Teskey HJ. Morphology and Terminology–Larvae In: Manual of Nearctic Diptera. Volume 1, Ottawa: Agriculture Canada; 1981 pp. 65–88.

[3] Grimaldi D, Engel MS. Evolution of the Insects 1st ed. Cambridge, New York, Melbourne: Cambridge University Press; 2005 p. 514.

[4] Sinclair BJ. A phylogenetic interpretation of the Brachycera (Diptera) based on the larval mandible and associated mouthpart structures. Syst Entomol. 1992;17: 233–252.

[5] Friedrich M, Tautz D. Evolution and phylogeny of the Diptera: a molecular phylogenetic analysis using 28s rDNA sequences. Syst Biol. 1997;674–698. 11975338 10.1093/sysbio/46.4.674

[6] De Meijere JHC. Zur Kenntnis des Kopfbaus der Dipteren Larven und Imagines. Zool Anz. 1916;46: 241–51.

[7] Cook F. The evolution of the head in the larvae of the Diptera. Microentomology 1949;14: 1–57.

[8] Rotheray GE, Gilbert F. Phylogenetic relationships and the larval head of the lower Cyclorrhapha (Diptera). Biol J Linn Soc. 2008;153: 287–323.

[9] Woodley NE. Phylogeny and classification of the "Orthorrhaphous" Brachycera In: Manual of Nearctic Diptera, Volume 3, Ottawa: Agriculture Canada; 1989 pp. 1371–1395.

[10] Chandler PJ. ed. A Dipterist's Handbook (2nd Edition). Oprington, Kent, England: The Amateur Entomologists' Society; 2010 pp. 442–467.

[11] Hernández MC. Biology of *Thrypticus truncatus* and *Thrypticus sagittatus* (Diptera: Dolichopodidae), Petiole miners of water hyacinth, in Argentina, with morphological descriptions of larvae and pupae. Ann Entomol Soc Am. 2008;101: 1041–1049.

[12] Mik J. Dipterologische Miscellen (2 Serie). Weiner Entomol Z. 1896;15: 241–278.

[13] Nartshuk EP. Taxonomic and faunistic data on the Rhagionidae (Diptera, Brachycera) of the northern Palaearctic. Acta Zool Fenn. 1995;17–24.

[14] Brindle A. Notes on the larvae of the British Rhagionidae and Stratiomyidae with a key to the genera. Entomol Rec. 1959;71: 126–133.

[15] Imada Y, Kato M. Bryophyte-feeding of *Litoleptis* (Diptera: Rhagionidae) with descriptions of new species from Japan. Zootaxa 2016;4097: 41–58. 10.11646/zootaxa.4097.1.2 27394524

[16] Mostovski MB, Jarzembowski EA, Coram R, Ansorge J. Curious snipe-flies of the genus *Ptiolinites* (Diptera: Rhagionidae) from the Purbeck of Dorset, the Wealden of the Weald and the Lower Cretaceous of Spain and Transbaikalia. Proc Geol Assoc. 2000;111: 153–160.

[17] Kovalev VG. The oldest representatives of the Diptera with short antennae from the Jurassic in Siberia. Paleontol J. 1981;15: 84–100.

[18] Kerr PH. Phylogeny and classification of Rhagionidae, with implications for Tabanomorpha (Diptera: Brachycera). Zootaxa 2010;2592: 1–133.

[19] Zhang J. New species of *Palaeobolbomyia* Kovalev and *Ussatchovia* Kovalev (Diptera, Brachycera, Rhagionidae) from the Callovian-Oxfordian (Jurassic) Daohugou biota of China: Biostratigraphic and paleoecologic implications. Geobios 2010;43: 663–669.

[20] Nagatomi A, Yang D. A review of extinct Mesozoic genera and families of Brachycera (Insecta, Diptera, Orthorrhapha). Ent mon Mag. 1998;134: 95–192.

[21] Roberts MJ. Structure of the mouthparts of the larvae of the flies *Rhagio* and *Sargus* in relation to feeding habits. J Zool. 1969;159: 381–398.

[22] Hobby BM, Smith KGV. The immature stages of *Chrysopilus cristatus* (F.) Entomol mon mag. 1961;97: 190–192.

[23] Krivosheina MG. Biology of Xylophilous larvae of the rhagionid flies *Chrysopilus dives* and *Chrysopilus nigrifacies* (Diptera, Rhagionidae). Entomol Rev. 2007;87: 1090–1097.

[24] Nagatomi A. The Japanese *Chrysopilus* (1). Mushi 1958;32: 33–41.

[25] Tsacas L. Recherches sur la structure et le fonctionnement de la tête et des pièces buccales larvaires de Rhagionidae (Diptères). Mém Mus Natl Hist Nat Ser A, Zool. 1962;27: 147–235.

[26] Sommerman KM. Alaskan snipe fly immatures and their habitat (Rhagionidae: *Symphoromyia*). Mosquito News 1962;22: 116–123.

[27] Brauer OF. Die Zweiflügler des Kaiserlichen Museums zu Wien. III. Systematische studein auf Grundlage der Dipteren-Larven nebst einer Zusammenstellung von Beispielen aus der Literatur über dieselben und Beschreibung neuer Formen. Kaiserl Akad Wiss Wien, Math Naturwiss Kl, Denkschr. 1883;47: 1–100.

[28] Lane RS, Anderson JR. Breeding sites of snipe flies (Rhagionidae) and other Diptera in woodland-grass soils. J Med Entomol. 1982;19: 104–108.

[29] Nagatomi A. The genera of Rhagionidae (Diptera). J Natur Hist. 1982;16: 31–70.

[30] Stuckenberg BR. Pruning the tree: a critical review of classifications of the Homeodactyla (Diptera, Brachycera), with new perspectives and an alternative classification. Stud Dipterol. 2001;8: 3–41.

[31] Wiegmann BM, Tsaur S-C, Webb DW, Yeates DK, Cassel BK. Monophyly and relationships of the Tabanomorpha (Diptera: Brachycera) based on 28S ribosomal gene sequences. Ann Entomol Soc Am. 2000;93, 1031–1038.

[32] Gibson JF, Kelso S, Jackson M, Kits J, Miranda GFG, Skevington JH. Novel, Diptera-specific PCR-amplification primers of use in molecular phylogenetic research. Ann Entomol Soc Am. 2011;104: 976–997.

[33] Tamura K, Stecher G, Peterson D, Filipski A, Kumar S. MEGA6: molecular evolutionary genetics analysis version 6.0. Mol Biol Evol. 2013;30: 2725–2729. 10.1093/molbev/mst197 24132122 PMC3840312

[34] Stamatakis A. RAxML Version 8: A tool for Phylogenetic Analysis and Post-Analysis of Large Phylogenies. Bioinformatics 2014;30(9): 1312–1313. 10.1093/bioinformatics/btu033 24451623 PMC3998144

[35] Ronquist F, Teslenko M, van der Mark P, Ayres DL, Darling A, Höhna S, et al MrBayes 3.2: efficient Bayesian phylogenetic inference and model choice across a large model space. Syst Biol. 2012;61: 539–542. 10.1093/sysbio/sys029 22357727 PMC3329765

[36] Schwarz G. Estimating the dimension of a model. Ann Statist. 1978;6: 461–464.

[37] Tanabe AS. Kakusan4 and Aminosan: two programs for comparing nonpartitioned, proportional, and separate models for combined molecular phylogenetic analyses of multilocus sequence data. Mol Ecol Resour. 2011;11: 914–921. 10.1111/j.1755-0998.2011.03021.x 21592310

[38] Rambaut A, Drummond AJ. Tracer v1.4 2007. In: Molecular evolution, phylogenetics and epidemiology [Internet]. Available: http://beast.bio.ed.ac.uk/Tracer. Accessed 13 October 2016.

[39] Ingrouille M, Eddie B. Plants: Diversity and Evolution. 1st ed. Cambridge: Cambridge University Press; 2006 pp. 197–198.

[40] Courtney GW, Sinclair BJ, Meier R. Morphology and terminology of Diptera larvae *In*: Papp L. & Darvas B. (Eds.) Contributions to a manual of Palaearctic Diptera, vol. 2 Science Herald, Budapest, 2000 pp. 85–161.

[41] Schremmer F. Morphologische und funktionelle Analyse der Mundteile und des Pharynx der Larve von *Stratiomys chamaeleon* L. Österr Zool Z. 1951;3: 326–397.

[42] Teskey HJ The immature stages and phyletic position of *Glutops rossi* (Diptera: Pelecorhynchidae). Canadian Entomol 1970;102: 1130–1135.

[43] Hennig W. Diptera (Zweiflügler) In. Helmcke J-G, Starck D, Wermuth H, editors. Handbuch der Zoologie. IV. Band: Arthropoda, 2. Hälfte: Insecta. 2 Walter de Gruyter, Berlin, New York; 1973 p. 564.

[44] Hinton HE. On the structure, function, and distribution of the prolegs of the Panorpoidea, with a criticism of the Berlese-Imms theory. Ecol Entomol. 1955;106: 455–540.

